# Odds Ratio or Prevalence Ratio? An Overview of Reported Statistical Methods and Appropriateness of Interpretations in Cross-sectional Studies with Dichotomous Outcomes in Veterinary Medicine

**DOI:** 10.3389/fvets.2017.00193

**Published:** 2017-11-10

**Authors:** Brayan Alexander Fonseca Martinez, Vanessa Bielefeldt Leotti, Gustavo de Sousa e Silva, Luciana Neves Nunes, Gustavo Machado, Luís Gustavo Corbellini

**Affiliations:** ^1^Laboratory of Veterinary Epidemiology, Faculty of Veterinary, Department of Preventive Veterinary Medicine, Universidade Federal do Rio Grande do Sul (UFRGS), Porto Alegre, Brazil; ^2^Faculty of Medicine, Department of Statistics, Institute of Mathematics and Statistics and Post-Graduate Program of Epidemiology, Universidade Federal do Rio Grande do Sul, Porto Alegre, Brazil

**Keywords:** odds ratio, prevalence ratio, veterinary epidemiology, log-binomial model, Bayesian model, cross-sectional study, Poisson model, logistic regression

## Abstract

One of the most commonly observational study designs employed in veterinary is the cross-sectional study with binary outcomes. To measure an association with exposure, the use of prevalence ratios (PR) or odds ratios (OR) are possible. In human epidemiology, much has been discussed about the use of the OR exclusively for case–control studies and some authors reported that there is no good justification for fitting logistic regression when the prevalence of the disease is high, in which OR overestimate the PR. Nonetheless, interpretation of OR is difficult since confusing between risk and odds can lead to incorrect quantitative interpretation of data such as “the risk is X times greater,” commonly reported in studies that use OR. The aims of this study were (1) to review articles with cross-sectional designs to assess the statistical method used and the appropriateness of the interpretation of the estimated measure of association and (2) to illustrate the use of alternative statistical methods that estimate PR directly. An overview of statistical methods and its interpretation using the Preferred Reporting Items for Systematic Reviews and Meta-Analyses (PRISMA) guidelines was conducted and included a diverse set of peer-reviewed journals among the veterinary science field using PubMed as the search engine. From each article, the statistical method used and the appropriateness of the interpretation of the estimated measure of association were registered. Additionally, four alternative models for logistic regression that estimate directly PR were tested using our own dataset from a cross-sectional study on bovine viral diarrhea virus. The initial search strategy found 62 articles, in which 6 articles were excluded and therefore 56 studies were used for the overall analysis. The review showed that independent of the level of prevalence reported, 96% of articles employed logistic regression, thus estimating the OR. Results of the multivariate models indicated that logistic regression was the method that most overestimated the PR. The findings of this study indicate that although there are methods that directly estimate PR, many studies in veterinary science do not use these methods and misinterpret the OR estimated by the logistic regression.

## Introduction

Cross-sectional studies are one of the most frequently designed observational studies in veterinary epidemiology (1), likely because they are rapid, inexpensive and of moderate difficulty. This type of design has been widely used in veterinary medicine to address a variety of research inquiries. Establishing a causal relationship between an exposure and outcome is not possible in these studies given that both are measured simultaneously, and therefore, to speak of risk is inadequate, since risk is the probability of an outcome in a population or the probability that a specific outcome or disease will develop during a specific period of time (2, 3). In this design, where binary outcomes (e.g., disease/not disease, positive/negative) are frequent, the odds ratio (OR) or prevalence ratio (PR) can be used as measures of association. However, some authors suggest the use of the PR because its interpretation is easier than the interpretation of the OR (4, 5).

In human epidemiology much has been discussed about the use of the OR exclusively for case–control studies and some authors encourage the use of PR for cross-sectional designs because the precise interpretation of the OR is difficult and often mistakenly interpreted as PR (4). Despite being mathematically identical to relative risk (RR), PR can only be used in cross-sectional studies and not in clinical trials or cohorts since the former measures prevalence instead of risk (6). It is noteworthy that leading journals in human health fields such as *New England Journal of Medicine* and the *American Journal of Epidemiology* have officially discouraged the use of the OR for any study in which other measures of association are ascertainable. Spiegelman and Hertzmark stated that “There is no longer any good justification for fitting logistic regression models and estimating odds ratios when the odds ratio is not a good approximation of the risk or prevalence ratio” (7).

The OR can be interpreted as the PR for rare outcomes, as they will be similar (8). Some authors even indicate that the OR is a good approximation of the PR and can be interpreted as such only when the outcome is rare along the two strata of exposure (9). When the binary outcome is common, usually with a prevalence greater than 10%, the PR can be overestimated by the OR when the PR is greater than 1 or underestimated when the PR is less than 1 (8, 10–14). Additionally, it has been reported that interpreting the OR as if it was a PR is inadequate not only in terms of the possible bias but also because confounding may not be appropriately controlled (14). Phrases including words such as “risk,” “more likely,” “likelihood,” or “probability” to interpret the OR are commonly found in the literature. These phrases are a risk-language, not odds-language, and it is important not to use them when the odds is the measure of disease frequency (8, 15, 16). It was stated that language like “X times as likely to” implies comparison of probabilities, not odds (17). Additionally, incorrect quantitative interpretation of data by confusing risk and odds, such as “OR = X, therefore the risk is X times greater” was reported (18).

One reason for the popularity of the OR is that it is directly estimated by the logistic regression, one of the statistical methods widely employed in the epidemiological literature (14, 19). Nevertheless, other alternatives methods to logistic regression that can estimate directly PR have been reported. One option is the log-binomial model, which is a generalized linear model with a binomial distribution and logarithmic link function (12, 14, 20, 21). Other options proposed are Poisson regression and Poisson regression with robust variance (11, 14). Poisson regression can estimate wide confidence intervals, and for that reason, a robust Poisson regression has been proposed (14, 22). Both models can eventually estimate probabilities greater than one, which is unrealistic (11). Problems of convergence have been described with log-binomial regression, especially when there are continuous independent variables. In this case, Poisson regression with robust variance should be used (7, 23, 24). The Bayesian approach for the log-binomial model has been proposed as an alternative when the frequentist log-binomial model presents convergence problems (25). Additionally, the Bayesian approach has been described as an interesting alternative when the outcome is polytomous or the data are correlated (i.e., has an hierarchical structure), which is common in veterinary medicine (9).

Although the issues exposed above have been widely discussed and identified primary in the human epidemiology literature, it is interesting to know if many observational studies in veterinary medicine are still using logistic regression and its OR estimation to interpret the PR even when there are statistical packages models that estimate PR directly. To our knowledge, few authors in veterinary science have explored and exemplified the issue concerning the interpretation of the OR in randomized trials and cohort studies (15, 26, 27).

Consistency is important for epidemiological studies, and thus it is necessary to establish consensus standards for analyzing and reporting results (28, 29) and also for the methods used as extensively discussed in medical literature (14, 21, 30). For this, examining epidemiological studies within the scope of the veterinary literature and discussing statistical methods to analyze data and its interpretation is needed. Therefore, the aims of this study were as follows: (1) to review articles that used cross-sectional studies among a range of peer-reviewed journals in veterinary science to assess both the statistical method employed and the appropriateness of the interpretation of the measure of association; (2) to illustrate the use of statistical methods that directly estimate the PR (log-binomial, Poisson, Poisson regression with robust variance and Bayesian approach for the log-binomial regression) using the results of a cross-sectional study carried out by our research group. Our hypothesis is that most of cross-sectional studies in veterinary would fit logistic regression models to estimate OR despite the prevalence of the disease and that incorrect interpretation of the association using risk-language would be reported. Therefore, this study intends to raise a discussion about alternatives models for estimating PR and the interpretation of the estimates.

## Materials and Methods

### Review Strategy

#### Search Strategy, Selection Criteria and Appraisal

An overview of statistical methods and its interpretation using the Preferred Reporting Items for Systematic Reviews and Meta-Analyses (PRISMA) guidelines (31), of the available literature was conducted using PubMed as the search engine and included articles that used a cross-sectional study design from January 1, 2013, to July 1, 2016. The expression “overview” is based on the definition of review types previously reported (32); here, we made a not exhaustive (i.e., not including all the journals) and comprehensive searching, in which an eligibility criteria was set, and analysis comprised the tabulation of the specifics information searched, as explained later in this section.

This review deemed a diverse set of peer-reviewed journals among the veterinary science field presented on the SCImago Journal Rank (SJR). Ten journals were selected based on whether their scope considers aspects such as methods and approaches in veterinary epidemiology, veterinary public health, prevention and management of infectious animal diseases (Table 1). Our hypothesis was that if we found that many articles in these Journals reported data analysis from cross-sectional studies using logistic regression and misinterpreted odds ratio as “risk,” the frequency of these findings would be equal or even worst compared to other Journals with lower impact factors.

**Table 1:** Peer-reviewed journals within the veterinary science field presented based on the SCImago Journal Rank (SJR).

Journal	SJR^a^
*Veterinary Parasitology*	1.213
*Journal of Veterinary Internal Medicine*	1.257
*Veterinary Microbiology*	1.381
*Preventive Veterinary Medicine*	1.265
*Transboundary and Emerging Diseases*	1.251
*American Journal of Veterinary Research*	0.888
*Zoonoses and Public Health*	1.276
*Theriogenology*	0.842
*BMC Veterinary Research*	0.952
*Tropical Animal Health and Production*	0.620

*^a^Data for the year 2015*.

Search syntax was designed using Boolean operators (AND, OR, and NOT), the name of the journal, keywords and year of publication for selecting items of specific interest. The search strategy identified only articles published in English language literature and those whose epidemiological design were cross sectional. It was decided *a priori* to exclude letters to editor, comments, and review articles. When we sought articles by only the abstract and keywords (described in the syntax for “Word Text”), the research was limited because a specific issue could not be written in the abstract and keywords. For that reason, “MeSH terms” were also used as it had the functionality of selecting articles sorted by terms. MeSH is a set of terms naming descriptors in a hierarchical structure that enables the search at various levels of specificity. The syntax used in the search strategy is available in the S1 Syntax in Supplementary Material. The process of screening and inclusion of the studies were made according to the PRISMA flow diagram.

From each article found using the search strategy, information about the prevalence of the disease, measure of association and statistical method employed was recorded. Two authors (Brayan Alexander Fonseca Martinez and Gustavo de Sousa e Silva) independently assessed the appropriateness of the methods according to the following criteria: (1) the interpretation of the measure of association estimated by the statistical method employed and (2) the statistical method used accordingly with the prevalence level.

Reviewers (Brayan Alexander Fonseca Martinez and Gustavo de Sousa e Silva) were advised to classify the interpretation of the OR and PR as inappropriate when it was interpreted using risk-language and was assumed as appropriate when it was interpreted as the ratio between odds for the OR or prevalence for the PR. The cut off for prevalence values was set at 10%. In the situation where the prevalence is greater than 10%, the OR estimated in logistic regression can overestimate the PR, as explained previously (8, 10, 11, 13, 14, 16). Therefore, models other than logistic regression are considered more appropriate. On the other hand, when the prevalence is less than 10%, the OR estimates will be closer to PR estimates. The full-length articles were reviewed in detail if the information needed was not adequate or clear in the abstracts.

Following the review, disagreements between the reviewers about the interpretations were solved by the evaluation of a biostatistician (Vanessa Bielefeldt Leotti) and a veterinarian epidemiologist (Luís Gustavo Corbellini). Inter-observer agreement between the reviewers about the number of articles with inconsistent interpretations of the measure of association estimated was quantified using the kappa statistic (33). This was calculated using an Excel (Microsoft Excel 2010) spreadsheet.

### Illustration of Models That Directly Estimate Prevalence Ratio in a Cross-sectional Study

#### Description of the Dataset Used

The dataset used to exemplify methods that directly estimate the PR comes from a cross-sectional study performed to estimate the herd prevalence of antibodies against bovine viral diarrhea virus (BVDV) in bulk tank milk in southern Brazil (available in the S2 Dataset in Supplementary Material). Samples were randomly selected from a population of 81,307 dairy herds in the state of Rio Grande do Sul, Brazil, wherein 388 herds were collected. More details about the sample design and BTM collection can be found elsewhere (34). A questionnaire was designed to gather information about the potential factors associated with BVDV transmission and/or its maintenance within a herd. It was applied during visits to the 388 selected herds in November 2013.

#### Multivariable Model

A robust Poisson multivariable model was built with the variables screened in the univariable analysis using data from a previously published study (33). The variables identified as significantly associated with BVDV (*p* < 0.05) in the main multivariable model were also used in four other models: log-binomial regression, logistic regression, Poisson regression, and Bayesian approach for log-binomial regression.

Denoting *Y_i_* as the dichotomous outcome indicating the BVDV serostatus of the *i*-th farm (1 for positive samples and 0 otherwise), and assuming that this outcome follows a binomial distribution, the model formulation to log-binomial regression is written as follows:

(1) $$\text{ln}(\pi_{i})=\beta_{0}+\beta_{1}X_{1i}+\beta_{2}X_{2i}\ldots +\beta_{k}X_{ki},$$

where π_i_ is the probability of the *i*-th farm being seropositive for BVDV, conditional on the independent variables X_1_, …, X*_k_*. β_0_ is the intercept and β_1_, …, β*_k_* are the coefficients for each independent variable. The model using Poisson regression is formulated as follows:

(2) $$\text{ln}(\lambda_{i})=\gamma_{0}+\gamma_{1}X_{1i}+\gamma_{2}X_{2i}\ldots +\gamma_{k}X_{ki},$$

where λ*_i_* is the mean of the *i*-th farm (in this case, the mean approximates the probability of being seropositive for BVDV) conditional on the independent variables X_1_, …, X*_k_*. γ_0_ is the intercept and γ_1_, …, γ*_k_* are the coefficients for each independent variable. Finally, the logistic regression is written as:

(3) $$\text{ln}\left(\frac{\pi_{i}}{1-\pi_{i}}\right)=\delta_{0}+\delta_{1}X_{1i}+\delta_{2}X_{2i}\ldots +\delta_{k}X_{ki},$$

where π_I_ is the probability of the *i*-th farm being seropositive for BVDV, conditional on the independent variables *X*_1_, …, *X_k_*. δ_0_ is the intercept and δ_1_, …, δ*_k_* are the coefficients for each independent variable.

All models were fitted to this dataset using SAS version 9.3 (SAS Institute, Cary, NC, USA) with PROC GENMOD, except for the Bayesian approach for the log-binomial regression (available in S3 Codes in Supplementary Material). The link function for logistic regression was *logit* and was *log* for Poisson regression and log-binomial regression. To specify the use of the robust variance estimator for the robust Poisson regression, the REPEATED statement was used (7). Both logistic and log-binomial regressions have the same binomial distribution for the outcome, while Poisson regression assumes Poisson distribution for the outcome. Predicted probabilities were obtained for each farm in the dataset using the PRED statement to check probabilities greater than one for the Poisson regression methods. The exponential of each regression coefficient and its confidence intervals were used as point and interval estimates for the OR for logistic regression and PR for the other models.

The Bayesian approach for the log-binomial model, with posterior distributions estimated by employing the Markov Chain Monte Carlo (MCMC) method, was performed using OpenBugs 3.2.2 (35) together with the R statistical environment [R Development Core Team (36)] and *BRugs* package (35) (available in S3 Codes in Supplementary Material). The *CODA* package (37) was used for summarizing and plotting the output from MCMC simulations. The prior distributions assigned to the model coefficients were normal with zero mean and variance 10^6^ with the addition of a restriction to prevent simulation of probabilities outside the interval [0,1] (25). To choose the number of interactions, burn-in period and thin for MCMC, graphical analysis and Gelman and Rubin statistic were used (38). The model was run with 50,000 iterations with the first 5,000 discarded as burn-in using three sample chains. The mode and the equal tails credible interval were used as Bayesian point and interval estimators, respectively.

For all methods, point estimates and 95% confidence/credible interval estimates were shown. For comparisons purposes between methods, the ranges of the confidence/credible intervals and the relative changes in point estimates were calculated (Δ%). For the point estimates, log-binomial regression was used as reference and the relative change with the other methods employed was calculated as follows:

(4) $$\begin{array}{c} \Delta \%=\text{100}\times \frac{\left(\text{point estimate by the method}-\text{Log-binomial point estimate}\right)}{\text{Log-binomial point estimate}}. \end{array}$$

The point estimate in log-binomial regression was chosen as reference value to compare the estimates produced by the other methods since some studies using simulated and observed data concluded that its estimates are more precise and accurate, and therefore, it would be the method of choice between the frequentist alternatives (12, 23, 24, 39).

## Results

### Literature Review

**Figure 1:**
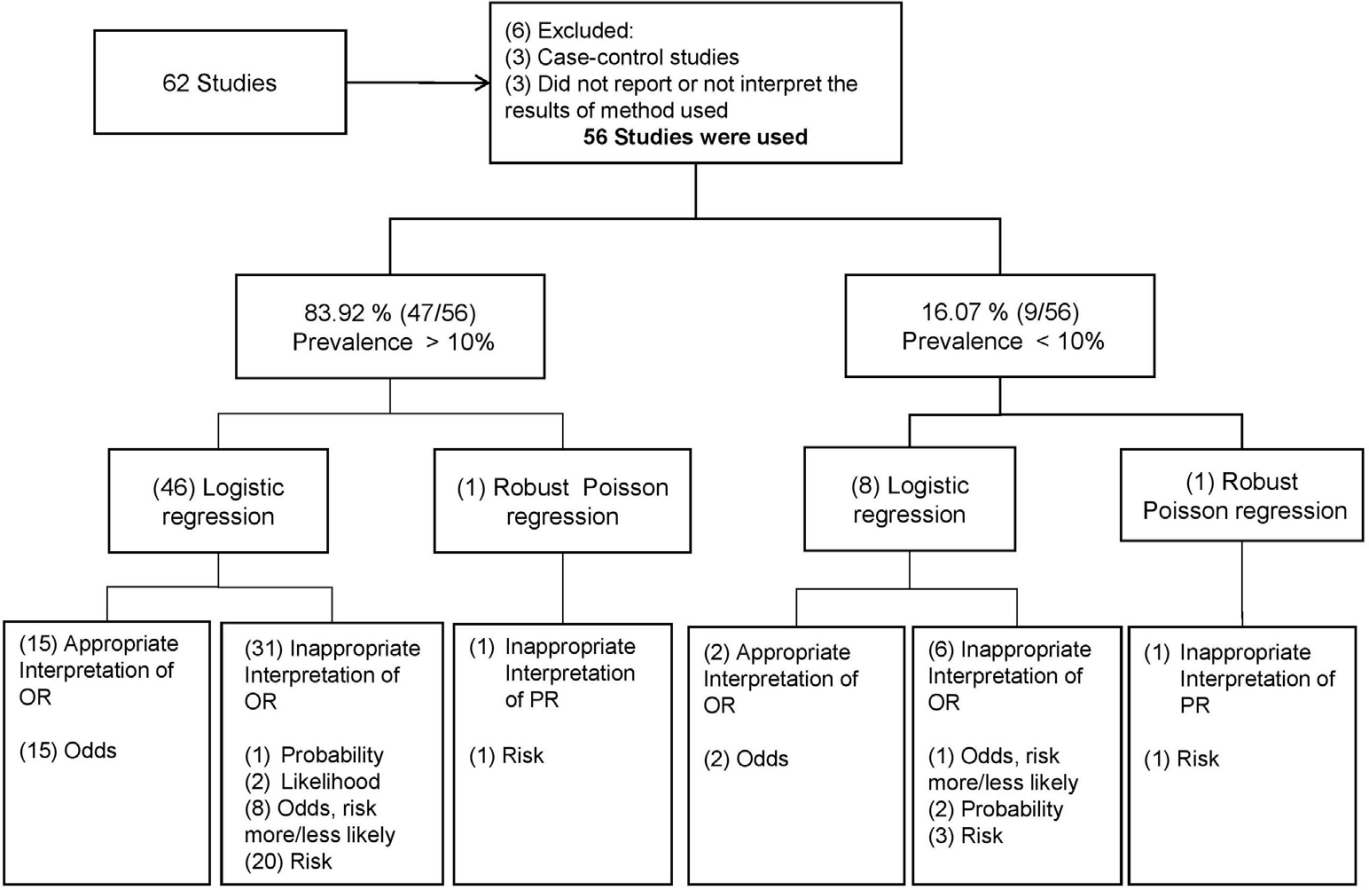
Results of the overview of reported statistical methods and their interpretations. The distribution of the number of articles by statistical methods and specific words used to interpret their estimates are within the brackets. The interpretation of the odds ratio (OR) and prevalence ratio (PR) was assumed as inappropriate when it was interpreted using words as “risk,” “(more/less) likely,” “probability,” or “likelihood” of the event and was assumed as appropriate when it was interpreted as the ratio between odds for the OR or prevalence for the PR.

The initial search strategy found 62 articles. Upon review of these abstracts, 6 articles were excluded for reasons outlined in Figure 1, and therefore 56 studies were used for the overall analysis (all articles reviewed are described in detail in the Table S1 in Supplementary Material; PRISMA Flow Diagram used is shown in Figure S1 in Supplementary Material). Once the biostatistician and the veterinarian epidemiologist resolved differences about interpretation and the statistical methods used in the studies, the review showed that 83.9% of the studies (47/56) reported prevalence values greater than 10% in the level of the outcome modeled (i.e., animal or herd level). Irrespective of the prevalence reported in the articles, logistic regression was described as the method for modeling the binary outcome in 96.4% (54/56) of the articles and only two described other methods, specifically robust Poisson regressions (Figure 1).

Regarding the interpretation of the measure of association, from the 47 articles with prevalence values greater than 10%, 15 of them made an appropriate interpretation of the OR as a ratio of *odds* or simply did not give a direct interpretation of the OR (Figure 1). Thirty-one articles reported the OR inappropriately using expression such as “risk” (*n* = 20), “likelihood” (*n* = 2), “probability” (*n* = 1), and “Odds and more/less likely” (*n* = 8). One article, despite reporting the PR estimated by a robust Poisson regression, presented an inappropriate interpretation of the PR as “risk.” Among the nine articles with prevalence values smaller than 10%, only two correctly interpreted the OR as a ratio of *odds*. Inappropriateness was found in six articles, since three interpreted the OR as “risk,” two reported the term “probability,” and one reported “Odds and more/less likely” interchangeable. One article, despite having estimated the PR, was interpreted inappropriately (Figure 1).

The two authors who reviewed the studies agreed on the appropriateness of the interpretation of the OR in 18 studies and agreed that 26 studies inappropriately interpreted the measure estimate based on the method used. The interobserved agreement, as measured by kappa statistics, was 0.6, showing a moderate agreement (33). Most of disagreement occurred in articles that reported terms such as “more/less likely.”

### Results from the Data Set Used as Example

The estimated prevalence of BVDV in the dataset used was 24% (CI 95%: 19.8–28.1). The final multiple robust Poisson regression identified three variables significantly associated with BVDV seropositivity (*p* < 0.05, Table 2). The results of the analyses using logistic, log-binomial, Poisson, and Bayesian log-binomial methods are also shown in Table 2. Regarding the relative changes observed between the methods used against the log-binomial regression, it can be observed that the point estimates in the logistic regression presented the largest differences ranging from 25.7 to 69.9%, whereas Poisson and Poisson regression with robust variance, which yielded the same point estimate as expected, had a difference ranging from 1.4 to 16.2%. The Bayesian approach for log-binomial regression had the lowest difference, ranging from 3.5 to 6.8%. For all the methods, logistic regression had wider intervals ranging from 2.19 to 4.97 (Table 2).

**Table 2:** Results for the three variables associated with the presence of antibodies against bovine viral diarrhea virus (BVDV) using different statistical methods.

Variable	Point (CI: 95%) and range by method							
	Logistic	Poisson	Robust Poisson	Log-binomial^b^	Bayesian log-binomial (MCMC)^a^	Δ_lgt_%^c^	Δ_poi_%^d^	Δ_bln_%^e^
Routine use of rectal examination	4.35 (2.52; 7.49) 4.97	2.82 (1.81; 4.39) 2.58	2.82 (1.96; 4.06) 2.09	2.56 (1.78; 3.67) 1.88	2.47 (1.78; 3.72) 1.94	69.91	10.15	−3.51
Direct contact over the fences	2.04 (1.22; 3.41) 2.19	1.64 (1.06; 2.54) 1.47	1.64 (1.13; 2.39) 1.26	1.62 (1.12; 2.34) 1.22	1.51 (1.14; 2.41) 1.27	25.93	1.23	−6.80
Farms that do not use artificial insemination (AI)	3.01 (1.60; 5.66) 4.06	2.07 (1.28; 3.34) 2.06	2.07 (1.41; 3.03) 1.62	1.78 (1.27; 2.49) 1.21	1.68 (1.21; 2.38) 1.17	69.10	16.30	−5.61

*^a^Credibility interval: 95%*.

*^b^Reference model*.

*^c^Relative difference in point estimates between the logistic and reference model*.

*^d^Relative difference in point estimates between the Poisson, robust Poisson, and Reference Model*.

*^e^Relative difference in point estimates between the Bayesian log-binomial, and reference model*.

As expected, the range of the intervals of the robust Poisson regression was less than those calculated by the Poisson regression. Except by the variable “farms that do not use artificial insemination,” the log-binomial regression had the narrowest intervals out of all the methods. In terms of statistical decision, no differences were found because the confidence intervals did not include the value 1; all variables were significantly associated with BVDV.

## Discussion

This study has several pertinent strengths and limitations. To our knowledge, this is the first study that assessed and reinforced the importance of the interpretation given to the measure of association estimated and the suitability of the statistical method used in cross-sectional studies published within the veterinary medicine. One limitation of this study is that the journals selected represent only a portion of the existing literature for a determined period. Therefore, these findings represent only the selected journals during a specific time-period and should not be extended to the whole universe of journals. The hypothesis was that if in the more recent publications from the selected journals that we assumed to publish articles within the scope of veterinary epidemiology we found mistakes of interpretation and the use of logistic regression in highly prevalent diseases, these results would be similar or even worst for older publications or in others Journals.

From the review performed in this study, only 3.5% (2/56) of the articles used some statistical method to directly estimate the measure of association indicated in cross-sectional studies, i.e., the PR, and all of them misinterpreted this measure of association. Moreover, the remaining 96.5% of the articles reported using a logistic regression to estimate the measure of association between the exposure and the binary outcome. This proportion contrasts with a similar search carried out online in 2003 that included highly reputable international journals of human epidemiology, in which logistic regression was used in 37 (34%) of the 110 cross-sectional studies (14). This vast majority of studies using logistic regression could lead to problems related to the interpretation given to the OR, and the overestimation/underestimation of the measure of association.

It is not inherently incorrect to report the OR in cross-sectional studies and its use does not incur in any problems if the authors interpret the OR as the ratio between odds or for rare diseases (14, 40). However, the majority of the articles (*n* = 37) reviewed interpreted the OR using sentences such as “Animals located on farm A have two times higher probability or risk of illness than other farm animals.” Sentences such as this are incorrect in cross-sectional studies for two reasons: the odds is not a ratio of probabilities or risks, and the cross-sectional design cannot evaluate risk, as the outcome and exposure are measured simultaneously. Perhaps the concepts of risk or likelihood are easier to understand than odds and for that reason it is common that the terms OR and RR/PR are treated interchangeably. Therefore, there is no guarantee that readers interpret the OR in the right way. This complexity with the interpretation of the OR was also evidenced by the moderate agreement between two authors about the appropriateness of the interpretation given to the OR, since they were instructed to report as inappropriate when they observe “risk-language,” which in fact have many expression such as “risk,” “likely,” “likelihood,” or “probability.” We attribute these differences to the use of confusing phrases such as “more/less likely” and “likely” to describe the OR. In fact, some authors have noted that the OR is difficult to understand and unintuitive (4, 41–43).

For example, a reasonable interpretation of the PR obtained for the variable “Direct contact over the fences” provided by the log-binomial regression in our example would be “Farms in which bovines have contact over fences with bovines from other farms had a prevalence of BVDV that was 0.62 times greater than farms in which bovines have no contact over fences with animals from other farms.” On the other hand, a good interpretation of the OR estimated by logistic regression for the same variable would be “Farms in which bovines have contact over fences with bovines from other farms had 1.04 times greater odds for BVDV than farms in which bovines have no contact over fences with animals from other farms.”

To overcome this problem of misinterpretation, it would be necessary and suitable to establish a pattern to prevent readers from making incorrect interpretations. In human health and veterinary research, Strengthening the Reporting of Observational Studies in Epidemiology (STROBE) and STROBE-Vet guidelines were developed to create homogeneity in the report of observational study results (29, 44). Authors are encouraged to use them to report observational study results. In this context, it would be important to alert researchers about the meaning of OR and PR and their interpretation avoiding to report estimates of cross-sectional studies using risk or probability-language.

The second problem is related to the overestimation or underestimation of the true association between exposure and outcome, as was observed in the example used in this study. In our review, from the 47 articles that reported a prevalence greater than 10%, 31 (66%) estimated OR and miss-interpreted them as risk language, consequently overestimating or underestimating the PR. On the other hand, in six articles wherein the OR was estimated, it could be approximated to the PR since the prevalence was lower than 10% (45).

Regarding this example used as illustration, the intervals produced by log-binomial regression were the narrowest, except for one variable; a simulation study has pointed that its confidence interval may be narrower than is true (12). As expected, the point estimates produced by the Poisson regression with and without robust variance were the same and distinct from the log-binomial regression, although some studies have reported that robust Poisson and log-binomial regression had similar results (22). It was expected that the confidence intervals obtained in Poisson regression would be wider than for log-binomial regression. This situation has been reported when the binary outcome is common (>10%), such as in our example, as a consequence of the fact that the Poisson distribution can have larger variance than the binomial distribution (11, 14). Moreover, the precision observed in the confidence intervals obtained by Poisson regression with robust variance was greater than Poisson regression due to the use of a sandwich estimator for the variances of the regression coefficients (22).

The well-recognized convergence problem using log-binomial regression when explanatory continuous variables are introduced (14, 46) was not observed here. To induce these problems, one continuous variable “total area of the farm dedicated to bovines” was added to the model, and convergence was not achieved (data not shown). The Bayesian approach was used to illustrate an alternative in cases where the convergence is a problem for the log-binomial method. The differences observed between Bayesian and frequentist approach for log-binomial regression were small, with relative change in point estimation ranging from 3.51 to 6.8%. Chu and Cole (25) reported that the inferences obtained by Bayesian and frequentist methods agree when the sample sizes are large and when a weak *a priori* distribution with respect to the information contained in the probability of observed data is used. Therefore, considering that prior distributions with minimal information were used, the slight differences observed could be attributed to the sample size and the MCMC simulation. Despite some studies using Poisson with variance robust regression reporting probabilities greater than one (47), all the predicted probabilities by this method in the example presented here were bound between 0 and 1.

The point estimate obtained by the logistic regression showed the greatest differences from all the other methods. Schiaffino et al. (45) reported that logistic regression overestimated the true measure of association, especially when the prevalence is greater than 10%, like the one used here (23.9%). This same study also reported that the confidence intervals provided by logistic regression were the widest in relation to other methods, as observed in our example. Therefore, this result reinforces the idea that logistic regression and its measure of association (OR) is only suitable for case–control studies and could approximate the PR only when the prevalence is smaller than 10%. Despite logistic regression estimations (i.e., OR) are distinct than the other models (i.e., PR), we compared the results of the point estimates and their intervals to illustrate how logistic regression estimates can be biased assuming that the PR is the most appropriated measure of association in cross-sectional studies. In other words, in many situation the reported impact of a given factor on the prevalence could be higher (or lower) than the reality if the results were based on the logistic regression models in a situation where the disease is common (i.e., >10%).

Given that the results of the studies and their interpretation are used as “raw material” for other processes such as systematic review, meta-analyses and quantitative risk assessment (28), it is important to provide reliable estimates and correct interpretation to avoid errors in medical decision making and even adverse public policy implications. Hence, excluding cross-sectional studies that reports improper measures of association, i.e., the ones that uses logistic regression in highly prevalent diseases and/or the ones reporting wrong interpretation could be a selection criterion when conducting a meta-analysis, for example.

In conclusion, to avoid possible inappropriate estimates and interpretations, the proper use of statistical techniques when conducting a cross-sectional study must be reinforced. Furthermore, it is important to standardize the interpretations of the measure of association, given the great confusion observed in the interpretations of the OR and PR. The use of prevalence ratio in cross-sectional studies should be encouraged since it is easier to interpret, also implying that logistic regression should not be used when the prevalence is high. Instead, log-binomial regression (frequentist or Bayesian approach) or robust Poisson regression should be used in these scenarios. Therefore, the key points toward improving the methodology applied in cross-sectional studies would be the following: (1) include in the guidelines of Journals the appropriateness of the methods for cross-sectional studies for a given prevalence level; (2) avoid using risk-language terms in cross-sectional studies, which are “risk,” “more/less likely,” “likelihood,” or “probability”; (3) prevalence ratio is the preferred measure of association in cross-sectional studies, which could be easily estimated with the advance of statistical softwares using the aforementioned models.

## Author Contributions

Conceptualization: LC and VL. Data curation: BM and GM. Formal analysis: BM, GS, VL, LN, and LC. Supervision: LC, VL, and LN. Writing—original draft preparation: BM.

## Conflict of Interest Statement

The authors declare that the research was conducted in the absence of any commercial or financial relationships that could be construed as a potential conflict of interest.
