# Supplementary material for: Odds Ratio or Prevalence Ratio? An Overview of Reported Statistical Methods and Appropriateness of Interpretations in Cross-sectional Studies with Dichotomous Outcomes in Veterinary Medicine
Source: Front Vet Sci. 2017 Nov 10;4:193. doi: 10.3389/fvets.2017.00193 (PMC5686058; doi:10.3389/fvets.2017.00193)
Supplement: Supplementary file 1 [file table_1.docx]

Supplementary Material

Odds ratio or prevalence ratio? An overview of reported statistical methods and appropriateness of interpretations in cross-sectional studies with dichotomous outcomes in veterinary medicine

**Brayan Alexander Fonseca Martinez, Vanessa Bielefeldt Leotti, Luciana Neves Nunes, Gustavo de Sousa e Silva, Gustavo Machado, Luís Gustavo Corbellini***

**Correspondence:** Luís Gustavo Corbellini: luis.corbellini@ufrgs.br, lgcorbellini@hotmail.com

# Supplementary Data

**S1 Syntax.** Syntax used in the systematic literature review

(cross sectional study[Text Word] OR cross sectional study[MeSH Terms] seroprevalence[MeSH Terms] OR seroepidemiologic studies[MeSH Terms] OR seroepidemiologic studies[Text Word] OR seroprevalence[Text Word] OR prevalence study[MeSH Terms] OR prevalence study[Text Word]) AND (odds ratio[MeSH Terms] OR odds ratio[Text Word] OR prevalence ratio[Text Word] prevalence[MeSH Terms] OR prevalence[Text Word] OR PR[Text Word] OR odds [Text Word]) AND (logistic model[MeSH Terms] OR logistic [Text Word] OR logistic regression[MeSH Terms] OR log-binomial [Text Word] OR poisson[Text Word]) AND ("Preventive veterinary medicine"[Journal] OR "BMC veterinary research"[Journal] OR "Tropical animal health and production"[Journal] OR "Theriogenology"[Journal] OR “Transboundary and Emerging Diseases”[Journal] OR "Zoonoses and public health"[Journal] OR "American journal of veterinary research"[Journal] OR " Journal of veterinary internal medicine / American College of Veterinary Internal Medicine "[Journal] OR "Veterinary microbiology"[Journal] OR “Veterinary Parasitology” [Journal] ) NOT (Editorial[ptyp] OR Letter[ptyp] OR Comment[ptyp] OR Review[ptyp])

**S2 Dataset.** Dataset used to test the multivariable models.

id bvd ai rectal contact

1 1 1 0 1

2 1 0 0 1

3 0 0 0 1

4 0 0 0 0

5 1 0 1 1

6 1 0 1 0

7 0 0 1 1

8 0 0 0 0

9 1 0 0 1

10 1 0 1 0

11 0 0 0 0

12 0 0 0 1

13 0 0 0 0

14 0 0 0 1

15 1 0 1 1

16 0 0 0 1

17 0 1 0 0

18 0 0 0 0

19 0 0 1 1

20 1 0 0 0

21 0 1 0 1

22 1 1 1 1

23 1 0 1 0

24 1 1 0 1

25 0 0 1 0

26 0 0 0 0

27 0 0 0 0

28 0 0 1 1

29 0 0 1 1

30 0 0 1 0

31 0 0 0 0

32 0 0 0 0

33 0 0 0 1

34 0 1 0 0

35 0 0 0 1

36 0 0 1 1

37 1 0 0 1

38 0 0 0 1

39 0 0 1 0

40 1 1 0 1

41 1 1 1 1

42 0 0 0 1

43 1 1 0 1

44 1 1 0 1

45 0 1 0 1

46 0 0 1 1

47 1 0 1 1

48 1 0 1 1

49 0 0 0 1

50 1 0 1 1

51 0 0 0 1

52 0 0 1 1

53 0 0 0 1

54 0 0 0 0

55 0 0 0 0

56 0 0 0 0

57 0 0 0 1

58 0 0 0 1

59 0 0 1 1

60 0 0 0 1

61 0 0 0 1

62 0 0 1 0

63 0 0 0 1

64 0 0 0 0

65 0 0 0 1

66 0 0 0 1

67 0 0 0 1

68 0 1 0 0

69 0 0 1 0

70 0 0 0 1

71 0 0 0 0

72 1 0 1 0

73 0 0 1 0

74 1 1 0 1

75 0 0 0 0

76 0 0 0 0

77 0 1 0 0

78 0 0 0 1

79 0 0 0 1

80 0 0 0 0

81 0 0 1 0

82 1 0 1 1

83 0 0 1 0

84 0 0 0 0

85 0 0 0 0

86 0 0 0 1

87 0 0 0 0

88 0 0 0 0

89 0 1 0 0

90 1 0 1 1

91 0 0 1 0

92 0 0 1 1

93 0 0 0 1

94 0 0 0 0

95 0 0 0 1

96 0 1 1 1

97 0 1 0 0

98 0 0 0 0

99 0 0 0 0

100 0 0 0 0

101 0 1 0 1

102 0 0 1 0

103 0 0 0 1

104 0 0 0 1

105 1 0 1 0

106 0 0 1 0

107 0 0 0 0

108 0 0 0 0

109 0 0 1 1

110 0 1 1 0

111 0 0 1 1

112 0 0 1 0

113 0 0 0 0

114 0 0 0 1

115 0 1 1 0

116 1 0 0 1

117 1 0 0 1

118 1 1 1 1

119 1 1 0 1

120 1 1 0 0

121 0 0 0 0

122 1 0 1 0

123 1 0 1 1

124 0 1 0 1

125 1 0 1 1

126 0 0 0 0

127 0 0 0 1

128 0 1 0 1

129 0 1 0 0

130 0 0 0 0

131 0 1 0 0

132 1 1 0 0

133 1 0 1 1

134 0 0 0 1

135 0 1 0 1

136 0 0 1 1

137 0 0 1 0

138 0 0 1 0

139 0 1 0 0

140 0 0 0 1

141 0 0 0 0

142 0 0 1 0

143 0 0 0 1

144 1 0 0 1

145 0 1 0 1

146 0 0 0 0

147 0 0 0 1

148 0 0 1 1

149 0 0 0 1

150 1 0 1 0

151 0 0 1 1

152 0 0 0 0

153 1 0 1 1

154 0 0 0 0

155 0 0 1 1

156 0 0 0 1

157 0 0 1 0

158 0 0 0 1

159 0 0 0 0

160 1 0 1 1

161 1 0 0 1

162 0 0 0 1

163 0 1 0 1

164 0 0 0 0

165 0 0 0 1

166 0 0 0 1

167 0 0 0 1

168 0 0 1 0

169 0 1 0 1

170 0 0 0 0

171 1 0 1 0

172 1 1 1 1

173 0 0 1 1

174 1 0 1 1

175 0 0 0 1

176 0 0 0 1

177 1 0 1 1

178 0 0 1 1

179 0 0 1 1

180 0 0 0 1

181 0 1 0 1

182 0 0 1 1

183 0 0 0 1

184 0 0 0 1

185 0 0 0 0

186 0 0 1 1

187 0 0 0 1

188 1 0 1 1

189 1 0 1 0

190 1 0 1 1

191 1 0 1 1

192 0 0 0 0

193 0 0 0 1

194 1 0 0 1

195 0 0 0 1

196 0 0 1 0

197 0 0 0 0

198 1 0 1 1

199 0 0 1 1

200 1 0 1 0

201 0 0 1 0

202 0 0 0 1

203 1 1 0 0

204 1 0 1 0

205 1 0 1 1

206 1 0 1 1

207 1 0 0 0

208 0 0 0 0

209 0 0 1 0

210 0 0 0 0

211 0 0 0 1

212 0 0 0 0

213 0 0 0 1

214 0 0 1 0

215 0 1 0 0

216 0 1 0 0

217 0 0 0 1

218 0 0 0 0

219 0 0 0 1

220 0 0 0 0

221 0 0 1 0

222 0 0 0 1

223 1 0 1 1

224 0 0 1 0

225 0 0 0 1

226 0 0 1 1

227 1 1 0 0

228 0 0 0 0

229 1 0 0 1

230 0 0 1 1

231 1 0 1 1

232 0 1 0 1

233 0 0 1 0

234 0 1 0 1

235 0 1 1 1

236 1 0 1 0

237 0 1 0 0

238 0 0 0 0

239 0 0 1 0

240 0 1 0 1

241 0 0 1 0

242 0 0 0 0

243 0 0 0 1

244 1 1 1 0

245 0 0 0 0

246 0 1 0 0

247 0 0 1 1

248 0 0 0 0

249 0 0 0 1

250 0 1 0 1

251 0 0 1 1

252 0 0 0 1

253 0 1 0 1

254 1 1 0 0

255 1 1 0 1

256 1 1 0 1

257 1 0 1 0

258 1 0 1 1

259 0 0 1 1

260 0 0 1 1

261 0 0 1 1

262 0 0 1 1

263 0 0 1 1

264 0 0 1 0

265 0 0 1 1

266 1 0 1 1

267 0 0 1 1

268 1 1 0 0

269 0 0 1 1

270 0 0 1 0

271 0 0 1 1

272 0 1 1 0

273 0 0 0 1

274 0 1 0 1

275 1 1 0 1

276 0 0 1 1

277 0 0 1 1

278 0 1 1 1

279 0 0 1 1

280 0 0 1 0

281 0 0 1 0

282 1 0 1 1

283 0 0 1 1

284 0 0 0 1

285 0 0 1 1

286 0 0 1 1

287 0 1 0 1

288 0 0 1 0

289 0 0 1 1

290 0 1 0 0

291 0 0 1 0

292 0 0 1 0

293 0 1 1 1

294 0 0 0 1

295 1 0 1 1

296 0 1 0 1

297 0 0 0 1

298 0 1 0 1

299 1 0 1 1

300 0 0 1 0

301 0 0 0 0

302 0 0 0 0

303 0 0 0 0

304 0 0 1 0

305 0 0 1 0

306 0 0 0 0

307 0 0 1 0

308 1 0 1 0

309 0 1 0 0

310 0 0 0 0

311 0 1 0 0

312 0 0 0 1

313 1 0 1 1

314 1 0 1 0

315 0 1 0 0

316 1 1 0 1

317 0 0 1 0

318 0 0 1 0

319 1 0 1 0

320 0 0 0 1

321 0 1 0 0

322 0 0 1 1

323 0 0 1 0

324 1 0 1 1

325 1 1 0 1

326 1 1 1 1

327 1 0 1 0

328 0 1 1 0

329 0 0 1 0

330 0 1 0 0

331 0 0 1 1

332 1 0 0 0

333 0 0 1 0

334 1 0 1 1

335 0 0 0 1

336 0 0 0 0

337 0 0 0 0

338 0 0 0 1

339 0 0 1 0

340 0 0 0 0

341 0 0 1 0

342 0 0 0 1

343 0 0 1 1

344 0 0 0 0

345 0 0 1 1

346 0 0 0 1

347 0 0 0 1

348 0 1 0 1

349 0 0 1

350 0 0 0 0

351 0 0 0 0

352 0 0 0 1

353 0 1 0 0

354 0 1 0 0

355 0 0 0 0

356 0 0 1 0

357 0 0 1 0

358 0 0 0 0

359 0 1 0 0

360 0 0 0 0

361 0 0 0 1

362 0 0 0 1

363 0 0 0 0

364 0 0 0 0

365 0 0 0 1

366 0 0 0 0

367 0 0 0 0

368 0 1 0 1

369 1 0 1 1

370 0 0 0 1

371 0 0 0 1

372 0 0 1 0

373 1 0 1 1

374 0 0 0 1

375 0 0 0 1

376 0 0 1 1

377 0 0 1 1

378 1 0 1 0

379 0 0 0 1

380 0 0 1 1

381 1 1 0 1

382 1 0 0 1

383 1 0 1 0

384 1 0 1 1

385 1 0 0 1

386 1 0 1 1

387 0 0 0 0

388 1 0 0 0

**S3 Codes.** SAS and R codes used in the multivariable models.

**Syntaxes used in SAS to perform logistic, log-binomial and Poisson regression:**

title 'logistic model’;

proc genmod desc;

class id ai (ref="1") /param=ref;

model bvd = ai rectal contact / dist=bin link=logit;

estimate 'contact over fence' contact 1-1/exp;

estimate 'rectal palpation' rectal 1-1/exp;

estimate 'artificial insemination' ai 1-1/exp;

output out=bvdv_logistico pred=PR1;

run;

title 'log-binomial ';

proc genmod desc;

class id ai (ref="1") (ref="1") /param=ref;

model bvd = ai rectal contact / dist=binary link=log;

estimate 'contact over fence' contact 1-1/exp;

estimate 'rectal palpation' rectal 1-1/exp;

estimate 'artificial insemination' ai 1-1/exp;

output out=bvdv_logbin pred=PR1;

run;

title 'robust Poisson regression';

proc genmod ;

class id ai (ref="1") /param=ref;

model bvd = ai rectal contact / dist=poisson link=log;

estimate 'contact over fence' contact 1-1/exp;

estimate 'rectal palpation' rectal 1-1/exp;

estimate 'artificial insemination' ai 1-1/exp;

output out=bvdv_poisson_rob pred=PR1;

repeated subject=id;

run;

**Syntax used in R to perform log-binomial Bayesian model:**

library(BRugs)

library(coda)

model <-read.csv("C:/file location/filename.csv", sep=",")

attach(model)

as.factor(ai)

ai=relevel(as.factor(ai), ref="1")

modelo = function (){

for(i in 1:n) {

Y[i] ~ dbern(p[i])

log(p[i]) <- alpha0 + alpha1 * X1[i] + alpha2 * X2[i] + alpha3 * X3[i]

}

#Prior

alpha1 ~dnorm(0,1.0E-6)

alpha2 ~dnorm(0,1.0E-6)

alpha3 ~dnorm(0,1.0E-6)

alpha0 ~dnorm(0,1.0E-6)

#OR

OR1 <- exp(alpha1)

OR2 <- exp(alpha2)

OR3 <- exp(alpha3)

}

#identifying parameters

parameters <- c("alpha0", "alpha1", "alpha2","alpha3", "OR1", "OR2", "OR3")

#dataset para BRugs

dados = list(n = dim(model)[1], Y = model$bvd, X1 = model$ai, X2 = model$rectal, X3 = model$contact)

#initial values

inits <- function(){

list(alpha0 =-10, alpha1=0, alpha2=0, alpha3=0)

list(alpha0 =-20, alpha1=0, alpha2=0, alpha3=0)

list(alpha0 =-30, alpha1=0, alpha2=0, alpha3=0)

}

# simulation

simul <- BRugsFit(modelFile = modelo, data= dados, inits, numChains = 1,parameters,nBurnin = 0, nIter=100, nThin=1, coda = T)

simul <- BRugsFit(modelFile = modelo, data= dados, inits, numChains = 3,parameters,nBurnin = 5000, nIter=30000, nThin=30, coda = T)

############ MODA OR1 ###############

kernel1 = density(as.matrix(simul[,' OR1']), from=min(as.matrix(simul[,' OR1'])), to=max(as.matrix(simul[,' OR1'])))

posmo1 = which.max(kernel1$y)

moda=kernel1$x[posmo1]

moda

############ MODA OR2 ###############

kernel1 = density(as.matrix(simul[,'OR2']), from=min(as.matrix(simul[,'OR2'])), to=max(as.matrix(simul[,'OR2'])))

posmo1 = which.max(kernel1$y)

moda=kernel1$x[posmo1]

moda

############ MODA OR3 ###############

kernel1 = density(as.matrix(simul[,'OR3']), from=min(as.matrix(simul[,'OR3'])), to=max(as.matrix(simul[,'OR3'])))

posmo1 = which.max(kernel1$y)

moda=kernel1$x[posmo1]

moda

################## Diagnostic ###########

plot(simul)

gelman.plot(simul)

autocorr.plot(simul)

autocorr.diag(simul)

effectiveSize(simul)

summary(simul)

# Supplementary Table

**Table 1.** Studies selected in a strategic literature review including articles published from January 1st, 2013 to July 1st, 2016 in journals in which their scope considers aspects such as methods and approaches in veterinary epidemiology, veterinary public health, prevention and management of infectious animal diseases.

| **N°** | **Statistical Method** | **Prevalence reported** | **Year** | **Reference** |
| --- | --- | --- | --- | --- |
| 1 | Logistic regression | Flock level prevalence: 28.3%; Animal level prevalence: 3.5 % | 2013 | (1) |
| 2 | Logistic regression | Brucella: 77.5%; Campylobacter: 31.2% | 2013 | (2) |
| 3 | Logistic regression | 35% | 2013 | (3) |
| 4 | Logistic regression | 71% | 2013 | (4) |
| 5 | Logistic regression | 22.5% | 2013 | (5) |
| 6 | Logistic regression | Herd level prevalence: 47.4%; Animal level prevalence 32.9% | 2013 | (6) |
| 7 | Logistic regression | Herd level prevalence: 66.5%; Animal level prevalence 35 % | 2013 | (7) |
| 8 | Logistic regression | Bulk tank milk prevalence: 2.5% | 2013 | (8) |
| 9 | Logistic regression | 54% | 2013 | (9) |
| 10 | Logistic regression | Prevalence Beef: 26%; Dairy: 62% | 2013 | (10) |
| 11 | Logistic regression | First case definition: 9%; Second case definition: 12% | 2013 | (11) |
| 12 | Logistic regression | 31.59% | 2013 | (12) |

| **Table 1.** (*Continued*) | | | | |
| --- | --- | --- | --- | --- |
| 13 | Logistic regression | 19% | 2013 | (13) |
| 14 | Logistic regression | Herd level prevalence: 11.4%; Animal level prevalence: 2.5% | 2013 | (14) |
| 15 | Logistic regression | Herd level prevalence: 95%; Animal level prevalence: 21.6% | 2013 | (15) |
| 16 | Logistic Regression | Prevalence at first and second sampling 17.4% and 27.4% | 2013 | (16) |
| 17 | Logistic Regression | *Leptospira*: 30.4 %; *C. burnetii*: 31.2 %; *Brucella*: 3.1 % | 2013 | (17) |
| 18 | Logistic Regression | *Salmonella*: 49%; *N. caninum*: 19%; *L. hardjo*: 86% | 2013 | (18) |
| 19 | Logistic Regression | Flock level prevalence: 11.2%; Animal level prevalence: 1.9% | 2013 | (19) |
| 20 | Logistic Regression | Sheep: 36%; Pigs: 4.7%; Deer: 6.6%; Chicken: 18% | 2013 | (20) |
| 21 | Logistic Regression | 71.3% | 2013 | (21) |
| 22 | Logistic Regression | Herd level prevalence: 14.5%; Animal level prevalence: 2.1% | 2014 | (22) |
| 23 | Logistic Regression | 1.3% | 2014 | (23) |
| 24 | Logistic Regression | 28,2% | 2014 | (24) |
| 25 | Logistic Regression | 85% | 2014 | (25) |
| 26 | Logistic Regression | Buffaloes: 43.3%; Cattle 28.6% | 2014 | (26) |
| 27 | Logistic Regression | 52.6% | 2014 | (27) |

| **Table 1.** (*Continued*) | | | | |
| --- | --- | --- | --- | --- |
| 28 | Logistic Regression | 24.50% | 2014 | (28) |
| 29 | Robust Poisson Regression | 33.8% | 2014 | (29) |
| 30 | Logistic Regression | 4.54% | 2014 | (30) |
| 31 | Logistic Regression | <0.1% | 2014 | (31) |
| 32 | Logistic Regression | Herd level prevalence: 4.1%; Animal level prevalence: 2% | 2014 | (32) |
| 33 | Robust Poisson Regression | 0.22% | 2014 | (33) |
| 34 | Logistic Regression | For all over disease >10% | 2014 | (34) |
| 35 | Logistic Regression | 27.7% | 2014 | (35) |
| 36 |  | *Not cross-sectional study | 2014 | (36) |
| 37 | Logistic Regression | 40.49% | 2014 | (37) |
| 38 | Logistic Regression | Herd level prevalence: 95.65%; Animal level prevalence: 31.9% | 2014 | (38) |
| 39 | Logistic Regression | Herd level prevalence: 48%; Animal level prevalence: 37% | 2014 | (39) |
| 40 | Logistic Regression | 41.60% | 2014 | (40) |
| 41 | Logistic Regression | 34.1% | 2014 | (41) |
| 42 | Logistic Regression | 47.1% in 2008 and 24.3% in 2009 | 2014 | (42) |
| **Table 1.** (*Continued*) | | | | |
| 43 | Logistic Regression | Herd level prevalence: 48.6%; Animal level prevalence: 14.7% | 2015 | (43) |
| 44 | Logistic Regression | Herd level prevalence: 63.2%; Animal level prevalence: 32.5% | 2015 | (44) |
| 45 | Logistic Regression | 12.3% | 2015 | (45) |
| 46 | Logistic Regression | 17.5% | 2015 | (46) |
| 47 | Logistic Regression | 18.1% | 2015 | (47) |
| 48 | Logistic Regression | Herd level prevalence: 95.65%; Animal level prevalence:31.9% | 2015 | (48) |
| 49 | Logistic Regression | 45% | 2015 | (49) |
| 50 | Logistic Regression | 20.3% | 2015 | (50) |
| 51 | Logistic Regression | 32.1% | 2015 | (51) |
| 52 | Logistic Regression | 40.9% | 2015 | (52) |
| 53 | Logistic Regression | 22 % | 2015 | (53) |
| 54 | Logistic Regression | 29.87% | 2015 | (54) |
| 55 | Logistic Regression | Calves, cows and dairy farms 12%, 12%, and 23% respectively; Animal level prevalence was 4% | 2015 | (55) |
| 56 | Logistic Regression | Herd level prevalence: 3.6%; Animal level prevalence: 2% | 2016 | (56) |
| 57 | Logistic Regression | *Brucella*: 9.2%; *Anaplasma*: 19.5%; *Babesia* 16% | 2016 | (57) |

| **Table 1.** (*Continued*) | | | | |
| --- | --- | --- | --- | --- |
| 58 | Logistic Regression | 44% | 2016 | (58) |
| 59 | Logistic Regression | 5.6% | 2016 | (59) |

# Suplemmentary Figure

**Figure 1.** Flowchart of the study selection process in this systematic review (PRISMA Flow Diagram).


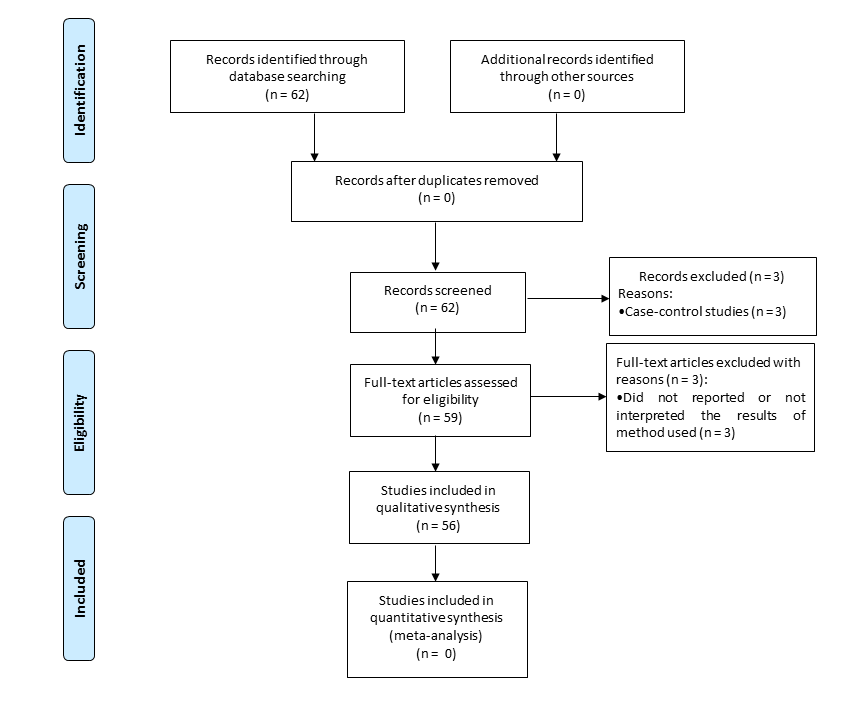


**References**

1. Teklue T, Tolosa T, Tuli G, Beyene B, Hailu B. Sero-prevalence and risk factors study of brucellosis in small ruminants in Southern Zone of Tigray Region, Northern Ethiopia. *Trop Anim Health Prod* (2013) **45**:1809–1815. doi:10.1007/s11250-013-0439-7

2. Mai HM, Irons PC, Kabir J, Thompson PN. Herd-level risk factors for Campylobacter fetus infection, Brucella seropositivity and within-herd seroprevalence of brucellosis in cattle in northern Nigeria. *Prev Vet Med* (2013) **111**:256–267. doi:10.1016/j.prevetmed.2013.05.016

3. Fablet C, Simon G, Dorenlor V, Eono F, Eveno E, Gorin S, Queguiner S, Madec F, Rose N. Different herd level factors associated with H1N1 or H1N2 influenza virus infections in fattening pigs. *Prev Vet Med* (2013) **112**:257–265. doi:10.1016/j.prevetmed.2013.07.006

4. Davlin S, Lapiz SM, Miranda ME, Murray K. Factors associated with dog rabies vaccination in Bhol, Philippines: results of a cross-sectional cluster survey conducted following the island-wide rabies elimination campaign. *Zoonoses Public Health* (2013) **60**:494–503. doi:10.1111/zph.12026

5. Rast L, Lee S, Nampanya S, Toribio J-ALML, Khounsy S, Windsor PA. Prevalence and clinical impact of Toxocara vitulorum in cattle and buffalo calves in northern Lao PDR. *Trop Anim Health Prod* (2013) **45**:539–546. doi:10.1007/s11250-012-0256-4

6. Sarrazin S, Veldhuis A, Meroc E, Vangeel I, Laureyns J, Dewulf J, Caij AB, Piepers S, Hooyberghs J, Ribbens S, et al. Serological and virological BVDV prevalence and risk factor analysis for herds to be BVDV seropositive in Belgian cattle herds. *Prev Vet Med* (2013) **108**:28–37. doi:10.1016/j.prevetmed.2012.07.005

7. Talafha AQ, Al-Majali AM. Prevalence and risk factors associated with Neospora caninum infection in dairy herds in Jordan. *Trop Anim Health Prod* (2013) **45**:479–485. doi:10.1007/s11250-012-0244-8

8. Pinho L, Thompson G, Machado M, Carvalheira J. Management practices associated with the bulk tank milk prevalence of Mycoplasma spp. in dairy herds in Northwestern Portugal. *Prev Vet Med* (2013) **108**:21–27. doi:10.1016/j.prevetmed.2012.07.001

9. Casais R, Goyena E, Martinez-Carrasco C, Ruiz de Ybanez R, Alonso de Vega F, Ramis G, Prieto JM, Berriatua E. Variable performance of a human derived Sarcoptes scabiei recombinant antigen ELISA in swine mange diagnosis. *Vet Parasitol* (2013) **197**:397–403. doi:10.1016/j.vetpar.2013.04.030

10. Gates MC, Woolhouse MEJ, Gunn GJ, Humphry RW. Relative associations of cattle movements, local spread, and biosecurity with bovine viral diarrhoea virus (BVDV) seropositivity in beef and dairy herds. *Prev Vet Med* (2013) **112**:285–295. doi:10.1016/j.prevetmed.2013.07.017

11. Zare Y, Shook GE, Collins MT, Kirkpatrick BW. Evidence of birth seasonality and clustering of Mycobacterium avium subspecies paratuberculosis infection in US dairy herds. *Prev Vet Med* (2013) **112**:276–284. doi:10.1016/j.prevetmed.2013.07.016

12. Gebremedhin EZ, Agonafir A, Tessema TS, Tilahun G, Medhin G, Vitale M, Di Marco V, Cox E, Vercruysse J, Dorny P. Seroepidemiological study of ovine toxoplasmosis in East and West Shewa Zones of Oromia Regional State, Central Ethiopia. *BMC Vet Res* (2013) **9**:117. doi:10.1186/1746-6148-9-117

13. Oseguera Montiel D, Frankena K, Udo H, Keilbach Baer NM, van der Zijpp A. Prevalence and risk factors for brucellosis in goats in areas of Mexico with and without brucellosis control campaign. *Trop Anim Health Prod* (2013) **45**:1383–1389. doi:10.1007/s11250-013-0375-6

14. Borba MR, Stevenson MA, Goncalves VSP, Neto JSF, Ferreira F, Amaku M, Telles EO, Santana SS, Ferreira JCA, Lobo JR, et al. Prevalence and risk-mapping of bovine brucellosis in Maranhao State, Brazil. *Prev Vet Med* (2013) **110**:169–176. doi:10.1016/j.prevetmed.2012.11.013

15. Bruhn FRP, Daher DO, Lopes E, Barbieri JM, da Rocha CMBM, Guimaraes AM. Factors associated with seroprevalence of Neospora caninum in dairy cattle in southeastern Brazil. *Trop Anim Health Prod* (2013) **45**:1093–1098. doi:10.1007/s11250-012-0330-y

16. Chaka H, Goutard F, Roger F, Bisschop SPR, Thompson PN. Household-level risk factors for Newcastle disease seropositivity and incidence of Newcastle disease virus exposure in backyard chicken flocks in Eastern Shewa zone, Ethiopia. *Prev Vet Med* (2013) **109**:312–320. doi:10.1016/j.prevetmed.2012.10.003

17. Mazeri S, Scolamacchia F, Handel IG, Morgan KL, Tanya VN, Bronsvoort BM deC. Risk factor analysis for antibodies to Brucella, Leptospira and C. burnetii among cattle in the Adamawa Region of Cameroon: a cross-sectional study. *Trop Anim Health Prod* (2013) **45**:617–623. doi:10.1007/s11250-012-0268-0

18. O’ Doherty E, Sayers R, O’ Grady L. Temporal trends in bulk milk antibodies to Salmonella, Neospora caninum, and Leptospira interrogans serovar hardjo in Irish dairy herds. *Prev Vet Med* (2013) **109**:343–348. doi:10.1016/j.prevetmed.2012.10.002

19. Asmare K, Megersa B, Denbarga Y, Abebe G, Taye A, Bekele J, Bekele T, Gelaye E, Zewdu E, Agonafir A, et al. A study on seroprevalence of caprine brucellosis under three livestock production systems in southern and central Ethiopia. *Trop Anim Health Prod* (2013) **45**:555–560. doi:10.1007/s11250-012-0258-2

20. Halova D, Mulcahy G, Rafter P, Turcekova L, Grant T, de Waal T. Toxoplasma gondii in Ireland: seroprevalence and novel molecular detection method in sheep, pigs, deer and chickens. *Zoonoses Public Health* (2013) **60**:168–173. doi:10.1111/j.1863-2378.2012.01514.x

21. Dias JA, Alfieri AA, Ferreira-Neto JS, Goncalves VSP, Muller EE. Seroprevalence and risk factors of bovine herpesvirus 1 infection in cattle herds in the state of Parana, Brazil. *Transbound Emerg Dis* (2013) **60**:39–47. doi:10.1111/j.1865-1682.2012.01316.x

22. Kazoora HB, Majalija S, Kiwanuka N, Kaneene JB. Prevalence of Mycobacterium bovis skin positivity and associated risk factors in cattle from western Uganda. *Trop Anim Health Prod* (2014) **46**:1383–1390. doi:10.1007/s11250-014-0650-1

23. Kirunda H, Erima B, Tumushabe A, Kiconco J, Tugume T, Mulei S, Mimbe D, Mworozi E, Bwogi J, Luswa L, et al. Prevalence of influenza A viruses in livestock and free-living waterfowl in Uganda. *BMC Vet Res* (2014) **10**:50. doi:10.1186/1746-6148-10-50

24. Trehy MR, German AJ, Silvestrini P, Serrano G, Batchelor DJ. Hypercobalaminaemia is associated with hepatic and neoplastic disease in cats: a cross sectional study. *BMC Vet Res* (2014) **10**:175. doi:10.1186/s12917-014-0175-x

25. Hering J, Hille K, Fromke C, von Munchhausen C, Hartmann M, Schneider B, Friese A, Roesler U, Merle R, Kreienbrock L. Prevalence and potential risk factors for the occurrence of cefotaxime resistant Escherichia coli in German fattening pig farms--a cross-sectional study. *Prev Vet Med* (2014) **116**:129–137. doi:10.1016/j.prevetmed.2014.06.014

26. Moore DP, Konrad JL, San Martino S, Reichel MP, Cano DB, Mendez S, Spath EJL, Odeon AC, Crudeli G, Campero CM. Neospora caninum serostatus is affected by age and species variables in cohabiting water buffaloes and beef cattle. *Vet Parasitol* (2014) **203**:259–263. doi:10.1016/j.vetpar.2014.04.011

27. Hussain MH, Saqib M, Raza F, Muhammad G, Asi MN, Mansoor MK, Saleem M, Jabbar A. Seroprevalence of Babesia caballi and Theileria equi in five draught equine populated metropolises of Punjab, Pakistan. *Vet Parasitol* (2014) **202**:248–256. doi:10.1016/j.vetpar.2014.01.026

28. Hireche S, Bouaziz O, Djenna D, Boussena S, Aimeur R, Kabouia R, Bererhi EH. Seroprevalence and risk factors associated with Chlamydophila spp. infection in ewes in the northeast of Algeria. *Trop Anim Health Prod* (2014) **46**:467–473. doi:10.1007/s11250-013-0515-z

29. Marks FS, Rodenbusch CR, Okino CH, Hein HE, Costa EF, Machado G, Canal CW, Brentano L, Corbellini LG. Targeted survey of Newcastle disease virus in backyard poultry flocks located in wintering site for migratory birds from Southern Brazil. *Prev Vet Med* (2014) **116**:197–202. doi:10.1016/j.prevetmed.2014.06.001

30. Paul S, Agger JF, Agerholm JS, Markussen B. Prevalence and risk factors of Coxiella burnetii seropositivity in Danish beef and dairy cattle at slaughter adjusted for test uncertainty. *Prev Vet Med* (2014) **113**:504–511. doi:10.1016/j.prevetmed.2014.01.018

31. Pecoraro HL, Bennett S, Huyvaert KP, Spindel ME, Landolt GA. Epidemiology and ecology of H3N8 canine influenza viruses in US shelter dogs. *J Vet Intern Med* (2014) **28**:311–318. doi:10.1111/jvim.12301

32. Lindahl E, Sattorov N, Boqvist S, Sattori I, Magnusson U. Seropositivity and risk factors for Brucella in dairy cows in urban and peri-urban small-scale farming in Tajikistan. *Trop Anim Health Prod* (2014) **46**:563–569. doi:10.1007/s11250-013-0534-9

33. Jackson DS, Nydam D V, Altier C. Prevalence and risk factors for brucellosis in domestic yak Bos grunniens and their herders in a transhumant pastoralist system of Dolpo, Nepal. *Prev Vet Med* (2014) **113**:47–58.

34. Yoak AJ, Reece JF, Gehrt SD, Hamilton IM. Disease control through fertility control: Secondary benefits of animal birth control in Indian street dogs. *Prev Vet Med* (2014) **113**:152–156. doi:10.1016/j.prevetmed.2013.09.005

35. Larska M, Krzysiak MK, Kesik-Maliszewska J, Rola J. Cross-sectional study of Schmallenberg virus seroprevalence in wild ruminants in Poland at the end of the vector season of 2013. *BMC Vet Res* (2014) **10**:967. doi:10.1186/s12917-014-0307-3

36. Delgado AH, Norby B, Scott HM, Dean W, McIntosh WA, Bush E. Distribution of cow-calf producers’ beliefs about reporting cattle with clinical signs of foot-and-mouth disease to a veterinarian before or during a hypothetical outbreak. *Prev Vet Med* (2014) **117**:505–517. doi:10.1016/j.prevetmed.2014.09.011

37. Gebremedhin EZ, Yunus HA, Tesfamaryam G, Tessema TS, Dawo F, Terefe G, Di Marco V, Vitale M. First report of Toxoplasma gondii in camels (Camelus dromedarius) in Ethiopia: bioassay and seroepidemiological investigation. *BMC Vet Res* (2014) **10**:222. doi:10.1186/s12917-014-0222-7

38. Song Q, Zhang W, Song W, Liu Z, Khan MK, He L, Fang R, Li P, Zhou Y, Hu M, et al. Seroprevalence and risk factors of Mycoplasma suis infection in pig farms in central China. *Prev Vet Med* (2014) **117**:215–221. doi:10.1016/j.prevetmed.2014.07.006

39. Gunawardana S, Thilakarathne D, Abegunawardana IS, Abeynayake P, Robertson C, Stephen C. Risk factors for bovine mastitis in the Central Province of Sri Lanka. *Trop Anim Health Prod* (2014) **46**:1105–1112. doi:10.1007/s11250-014-0602-9

40. Jiang Q, Zhou J, Jiang Z, Xu B. Identifying risk factors of avian infectious diseases at household level in Poyang Lake region, China. *Prev Vet Med* (2014) **116**:151–160. doi:10.1016/j.prevetmed.2014.04.016

41. Rooney AL, Limon G, Vides H, Cortez A, Guitian J. Sarcocystis spp. in llamas (Lama glama) in Southern Bolivia: a cross sectional study of the prevalence, risk factors and loss in income caused by carcass downgrades. *Prev Vet Med* (2014) **116**:296–304. doi:10.1016/j.prevetmed.2013.11.014

42. Rossi S, Pioz M, Beard E, Durand B, Gibert P, Gauthier D, Klein F, Maillard D, Saint-Andrieux C, Saubusse T, et al. Bluetongue dynamics in French wildlife: exploring the driving forces. *Transbound Emerg Dis* (2014) **61**:e12-24. doi:10.1111/tbed.12061

43. Meadows S, Jones-Bitton A, McEwen S, Jansen J, Menzies P. Coxiella burnetii seropositivity and associated risk factors in sheep in Ontario, Canada. *Prev Vet Med* (2015) **122**:129–134. doi:10.1016/j.prevetmed.2015.07.007

44. Meadows S, Jones-Bitton A, McEwen S, Jansen J, Menzies P. Coxiella burnetii seropositivity and associated risk factors in goats in Ontario, Canada. *Prev Vet Med* (2015) **121**:199–205. doi:10.1016/j.prevetmed.2015.06.014

45. Castillo-Neyra R, Chou Chu L, Quispe-Machaca V, Ancca-Juarez J, Malaga Chavez FS, Bastos Mazuelos M, Naquira C, Bern C, Gilman RH, Levy MZ. The potential of canine sentinels for reemerging Trypanosoma cruzi transmission. *Prev Vet Med* (2015) **120**:349–356. doi:10.1016/j.prevetmed.2015.04.014

46. Sawford K, do Karmo A, da Conceicao F, Geong M, Tenaya IWM, Hartawan DHW, Toribio J-ALML. An investigation of classical swine fever virus seroprevalence and risk factors in pigs in Timor-Leste. *Prev Vet Med* (2015) **122**:99–106. doi:10.1016/j.prevetmed.2015.09.012

47. Musallam II, Abo-Shehada M, Omar M, Guitian J. Cross-sectional study of brucellosis in Jordan: Prevalence, risk factors and spatial distribution in small ruminants and cattle. *Prev Vet Med* (2015) **118**:387–396. doi:10.1016/j.prevetmed.2014.12.020

48. Tornimbene B, Frossard J-P, Chhim V, Sorn S, Guitian J, Drew TW. Emergence of highly pathogenic porcine reproductive and respiratory syndrome (HP-PRRS) in medium-scale swine farms in southeastern Cambodia. *Prev Vet Med* (2015) **118**:93–103. doi:10.1016/j.prevetmed.2014.08.009

49. Ndhlovu DN, Masika PJ. Risk factors associated with clinical dermatophilosis in smallholder sector cattle herds of Zimbabwe at the Amblyomma variegatum and Amblyomma hebraeum interface. *Trop Anim Health Prod* (2015) **47**:353–360. doi:10.1007/s11250-014-0727-x

50. Yin M-Y, Wang J-L, Huang S-Y, Qin S-Y, Zhou D-H, Liu G-X, Tan Q-D, Zhu X-Q. Seroprevalence and risk factors of Toxoplasma gondii in Tibetan Sheep in Gansu province, Northwestern China. *BMC Vet Res* (2015) **11**:41. doi:10.1186/s12917-015-0358-0

51. Gebremedhin EZ, Kebeta MM, Asaye M, Ashenafi H, Di Marco V, Vitale M. First report on seroepidemiology of Toxoplasma gondii infection in pigs in Central Ethiopia. *BMC Vet Res* (2015) **11**:59. doi:10.1186/s12917-015-0384-y

52. Wesonga FD, Gachohi JM, Kitala PM, Gathuma JM, Njenga MJ. Theileria parva infection seroprevalence and associated risk factors in cattle in Machakos County, Kenya. *Trop Anim Health Prod* (2015) **47**:93–101. doi:10.1007/s11250-014-0690-6

53. Angwech H, Nyeko JHP, Opiyo EA, Okello-Onen J, Opiro R, Echodu R, Malinga GM, Njahira MN, Skilton RA. Heterogeneity in the prevalence and intensity of bovine trypanosomiasis in the districts of Amuru and Nwoya, Northern Uganda. *BMC Vet Res* (2015) **11**:1–8. doi:10.1186/s12917-015-0567-6

54. Kardjadj M, Kouidri B, Metref D, Luka PD, Ben-Mahdi MH. Seroprevalence, distribution and risk factor for peste des petits ruminants (PPR) in Algeria. *Prev Vet Med* (2015) **122**:205–210. doi:http://dx.doi.org/10.1016/j.prevetmed.2015.09.002

55. Tarazi YH, Abo-Shehada MN. Herd- and individual-level prevalences of and risk factors for Salmonella spp. fecal shedding in dairy farms in Al-Dhulail Valley, Jordan. *Trop Anim Health Prod* (2015) **47**:1241–1248. doi:10.1007/s11250-015-0854-z

56. Correa-Valencia NM, Ramirez NF, Olivera M, Fernandez-Silva JA. Milk yield and lactation stage are associated with positive results to ELISA for Mycobacterium avium subsp. paratuberculosis in dairy cows from Northern Antioquia, Colombia: a preliminary study. *Trop Anim Health Prod* (2016) **48**:1191–1200. doi:10.1007/s11250-016-1074-x

57. Lolli C, Marenzoni ML, Strona P, Lappo PG, Etiang P, Diverio S. Infections and risk factors for livestock with species of Anaplasma, Babesia and Brucella under semi-nomadic rearing in Karamoja Region, Uganda. *Trop Anim Health Prod* (2016) **48**:603–611. doi:10.1007/s11250-016-1005-x

58. Tsegay K, Potts AD, Aklilu N, Lotter C, Gummow B. Circulating serovars of Leptospira in cart horses of central and southern Ethiopia and associated risk factors. *Prev Vet Med* (2016) **125**:106–115. doi:10.1016/j.prevetmed.2016.01.009

59. Vilibic-Cavlek T, Vilibic M, Kolaric B, Jemersic L, Kucinar J, Barbic L, Bagaric A, Stevanovic V, Tabain I, Sviben M, et al. Seroepidemiology of Hepatitis E in Selected Population Groups in Croatia: A Prospective Pilot Study. Zoonoses Public Health (2016) 63:494–502. doi:10.1111/zph.12254
